# Altered microRNA profiles in cerebrospinal fluid exosome in Parkinson disease and Alzheimer disease

**DOI:** 10.18632/oncotarget.6158

**Published:** 2015-10-19

**Authors:** YaXing Gui, Hai Liu, LiShan Zhang, Wen Lv, XingYue Hu

**Affiliations:** ^1^ Department of Neurology, Sir Run Run Shaw Hospital, Affiliated with School of Medicine, Zhejiang University, Hangzhou, Zhejiang, China

**Keywords:** Parkinson's diseases (PD), Alzheimer's diseases (AD), cerebrospinal fluid (CSF), exosome, microRNA, Pathology Section

## Abstract

The differential diagnosis of Parkinson's diseases (PD) is challenging, especially in the early stages of the disease. We developed a microRNA profiling strategy for exosomal miRNAs isolated from cerebrospinal fluid (CSF) in PD and AD. Sixteen exosomal miRNAs were up regulated and 11 miRNAs were under regulated significantly in PD CSF when compared with those in healthy controls (relative fold > 2, *p* < 0.05). MiR-1 and miR-19b-3p were validated and significantly reduced in independent samples. While miR-153, miR-409-3p, miR-10a-5p, and let-7g-3p were significantly over expressed in PD CSF exosome. Bioinformatic analysis by DIANA-mirPath demonstrated that Neurotrophin signaling, mTOR signaling, Ubiquitin mediated proteolysis, Dopaminergic synapse, and Glutamatergic synapse were the most prominent pathways enriched in quantiles with PD miRNA patterns. Messenger RNA (mRNA) transcripts [amyloid precursor protein, APP), α-synuclein (α-syn), Tau, neurofilament, light gene (NF-L), DJ-1/PARK7, Fractalkine and Neurosin] and long non-coding RNAs (RP11-462G22.1 and PCA3) were differentially expressed in CSF exosomes in PD and AD patients. These data demonstrated that CSF exosomal RNA molecules are reliable biomarkers with fair robustness in regard to specificity and sensitivity in differentiating PD from healthy and diseased (AD) controls.

## INTRODUCTION

Parkinson's disease (PD) is a progressive disorder of the nervous system affecting approximately 1-2% of individuals over 60 years of age [1]. PD affects the nerve cells in the brain that produce dopamine and its symptoms include muscle rigidity, tremors, and changes in speech and gait [1]. Alzheimer's disease (AD) accounts for 60% to 70% of cases of dementia. It is a chronic neurodegenerative disease that usually starts slowly and gets worse over time [2]. Histopathologically, the AD brain is characterized by deposition of both neuritic plaques composed of amyloid-β (Aβ) peptide and hyper phosphorylated forms of the microtubule-associated protein Tau that create neurofibrillary tangles [3]. Clinical diagnosis of PD and AD is difficult in early stages of disease, with high risk of misdiagnosis. Reliable biomarkers are required for diagnosis of these neurodegenerative diseases, and tracking the disease progression.

As molecular changes in the brain are reflected in cerebrospinal fluid (CSF) composition, the CSF represents an optimal source of biomarkers of neurodegenerative diseases [4]. Studies in PD have highlighted the utility of CSF biomarkers in early diagnosis. For example, amyloid-β_1-42_ (Aβ_42_), total tau (t-tau) and phosphorylated tau (p-tau) are known markers of AD disease, in that they reliably reflect AD pathology [5]. In addition, α-synuclein (α-syn) induces aggregation and polymerization of tau, which promotes formation of abundant intracellular amyloid-tau inclusions [6]. Moreover, co-occurrence of tau and α-syn pathology has been detected in the neurons under various neurodegenerative diseases, such as AD and PD [7]. In addition, CSF measurement of DJ1/PARK7, a multifunctional protein implicated in the response to oxidative stress during the process of neurodegeneration, was decreased in PD [8]. The sensitivity and specificity, however, appear to be only moderate, and no correlation with PD severity or progression has been observed [8, 9]. The ability to meaningfully profile cerebrospinal fluid would gain insights about the underlying severity of central nervous system pathology.

Exosomes are present in most body fluids including saliva, plasma, and breast milk [10]. Exosomes can transfer genetic material to nearby cells, thereby affecting the function of the recipient cell [10-12]. However, the importance of exosomes in the pathogenesis of neurodegeneration *in vivo* has yet been established. MicroRNAs (miRNAs) are small (22-nt) endogenous noncoding RNAs that anneal to the 3′UTR of target mRNAs to mediate inhibition of translation [13]. More importantly, abnormal expression of miRNAs have been detected in AD and PD [14, 15]. However, alterations of exosomal miRNA content in CSF from PD and AD patients have not yet been described. The primary goal of this study was to characterize differential expression in exosomal miRNAs in PD and AD, and to explore their potential as biomarkers in AD and PD.

## RESULTS

### FACS characterization of CSF exosomal phenotypes

To confirm that the structures studied indeed are exosomal phenotyped vesicles, they were examined by flow cytometric analysis. CSF was treated with Dynabeads to detect surface CD63. Pilot experiments showed the identity of CSF vesicles was confirmed as exosomes by FACS analysis by specifically binding to latex beads coated with anti-CD63 (Figure S1A), which demonstrated the presence of the surface protein CD63, a commonly used marker of exosomes. Further analysis indicated that exosomes from all samples showed the presence of MHCII. However, MHC class I, CD54, and CD86 were not detected (data not shown). No significant differences were seen between groups. We further confirm the structures studied indeed are exosomes, they were examined by electron microscopy (Figure S1B). The electron micrographs of the exosomes revealed rounded structures with a size of approximately 50-80 nm, similar to previously described exosomes.

### MiRNAs were differentially expressed in CSF exosomes in PD and AD patients

**Figure 1:**
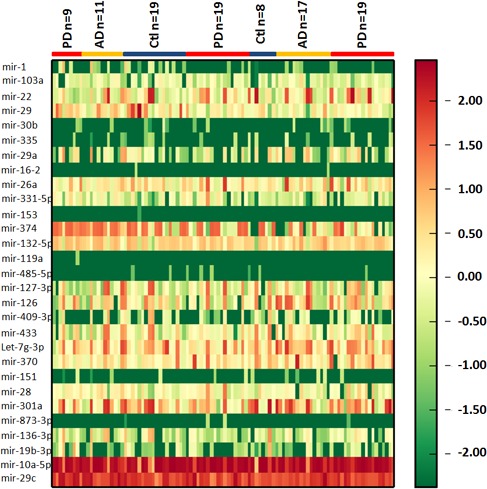
Heatmap of CSF exosomal differential miRNA profiles in PD and AD Heatmap representation of the mean fold change in PD and AD related differential miRNA signature. Two-dimensional grid matrix displaying 27 exosomal miRNAs in CSF was obtained by the functional heat-map in R. Columns refer to time course comparison: 27 healthy controls, 28 AD patients and 47 PD patients. Rows stand for the 27 differential miRNAs. Each entry of the grid refers to relative fold (log2) between the expression level of a given miRNA in exosome relative to RNU6B in healthy controls. The color of each entry is determined by the value of that fold difference, ranging from green (negative values) to red (positive values).

In an initial effort to identify differentially expressed exosomal miRNA in CSF of PD and AD patients, we profiled the expression of 746 miRNAs by using TaqMan miRNA arrays. To investigate the relative abundances of the exosome miRNAs detected, they were normalized in each sample to RNU6B. The data indicated that 132 miRNAs (17.7%) could be detected (assays giving Ct < 35, miRNA present in one of three groups was classed as detectable). The study revealed differential expression of 27 exosomal miRNAs in PD CSF compared to those in healthy controls. Among them, we found that 16 exosomal miRNAs were up regulated and 11 miRNAs were under regulated significantly (*p* < 0.05) in CSF from PD patients when compared with healthy controls (Figure 1 and Table 2). However, only mir-29c, mir-136-3p, mir-16-2, mir-331-5p, mir-132-5p, and mir-485-5p were significantly changed in AD CSF compared to those in healthy controls (Table 2). In addition, the plots for disease phenotypes (healthy, PD and AD) were performed as principal component analysis (PCA) among all samples based on miRNA profiles (Figure S2). Healthy and AD was not correlated with the first and second principal components. PD was correlated with the first PC (*p* = 0.005), which suggested that the statistical results from differential miRNA expression profiling would be affected by principal components when testing differential exosomal miRNAs expression. Taken all together, these data indicated that miRNAs were present in CSF exosomes, and exosomal miRNAs were differentially expressed in PD and AD patients relative to healthy controls.

**Table 1:** Demographics of study participants and CSF marker levels in diagnostic groups

Clinical features/Diseases	Healthy controls	Alzheimer's disease	Parkinson's disease
Number of subjects	27	28	47
Age (years)	60±13(42-79)	65±12(40-78)	63±9(45-77)
Gender (F/M)	8/9	13/15	22/25
Duration of disease (months)	..	33±16(15-70)	138±79(29-306)
CSF total protein (μg/μl)	0.88±0.50(0.23-1.98)	0.77±0.21(0.19-1.17)	1.05±0.37(0.55-3.25)
CSF a-synuclein (pg/μl)	0.98±0.49(0.32-2.08)	1.88±1.22(0.45-8.22)	1.22±0.77(0.44-4.55)
CSF tau protein (pg/ml)	177±97(78-305)	877±234(144-1982)	204±102(77-768)
DJ-1 (ng/ml)	44±22(17-88)	40±15(19-81)	31±12(14-55)

Data shown are mean±SD.

**Table 2:** Differential exosomal miRNA expression in CSF in AD and PD patients

PD vs. Control			AD vs. Control		
has-mirName	FC (log2)	Adjusted p-value	has-mirName	FC (log2)	Adjusted p-value
hsa-mir-1	−1.56	0.0078	hsa-mir-1	−0.28	0.1189
hsa-mir-103a	1.02	0.0084	hsa-mir-103a	0.12	0.0920
hsa-mir-22	−0.97	0.0090	hsa-mir-22	0.70	0.0519
hsa-mir-29	−0.69	0.0047	hsa-mir-29	−0.60	0.9064
hsa-mir-30b	1.22	0.0044	hsa-mir-30b	0.79	0.1518
hsa-mir-16-2	0.77	0.0039	hsa-mir-16-2	−0.83	0.0136
hsa-mir-26a	1.11	0.0058	hsa-mir-26a	0.41	0.9700
hsa-mir-331-5p	0.77	0.0082	hsa-mir-331-5p	−0.61	0.0404
hsa-mir-153	1.33	0.0057	hsa-mir-153	0.47	0.5501
hsa-mir-374	−0.56	0.0095	hsa-mir-374	−0.04	0.5982
hsa-mir-132-5p	0.89	0.0023	hsa-mir-132-5p	0.12	0.0188
hsa-mir-119a	−0.39	0.0061	hsa-mir-119a	−0.56	0.6787
hsa-mir-485-5p	1.22	0.0025	hsa-mir-485-5p	1.39	0.0269
hsa-mir-127-3p	2.33	0.0035	hsa-mir-127-3p	−0.27	0.6164
hsa-mir-126	−1.13	0.0038	hsa-mir-126	−0.76	0.2083
hsa-mir-409-3p	1.52	0.0039	hsa-mir-409-3p	0.37	0.0558
hsa-mir-433	2.05	0.0043	hsa-mir-433	0.06	0.8512
hsa-mir-370	2.73	0.0069	hsa-mir-370	0.31	0.0824
hsa-let-7g-3p	0.63	0.0068	hsa-let-7g-3p	0.42	0.8608
hsa-mir-151	−0.88	0.0073	hsa-mir-151	0.51	0.0063
hsa-mir-28	−0.17	0.0035	hsa-mir-28	−0.42	0.6606
hsa-mir-301a	−0.48	0.0054	hsa-mir-301a	−0.51	0.0650
hsa-mir-873-3p	1.63	0.0052	hsa-mir-873-3p	0.70	0.6384
hsa-mir-136-3p	0.74	0.0068	hsa-mir-136-3p	−0.12	0.0133
hsa-mir-19b-3p	−0.93	0.0109	hsa-mir-19b-3p	0.38	0.9713
hsa-mir-10a-5p	0.65	0.0017	hsa-mir-10a-5p	0.14	0.3582
hsa-mir-29c	−0.44	0.0013	hsa-mir-29c	−0.47	0.0436

### Comparative pathway analyses

In order to examine which biologic pathways were affected by differential exosomal miRNAs, we applied DIANA-mirPath on PD related dysregulated exosmal miRNA signature for further investigation. Forty-two KEGG biological processes were significantly enriched (*p* < 0.05, FDR corrected) among differentially expressed exosomal miRNAs in PD CSF when compared with those of healthy controls. Among them, Neurotrophin signaling pathway (*p* = 5.70E-13), mTOR signaling pathway (*p* = 2.86E-12), Ubiquitin mediated proteolysis (*p* = 2.86E-12), Long-term potentiation (*p* = 3.68E-12), Axon guidance (p = 3.51E-11), Cholinergic synapse (*p* = 1.75E-10), Gap junction (*p* = 7.09E-10), Dopaminergic synapse (*p* = 3.43E-08), Glutamatergic synapse (*p* = 1.76E-05) were the most prominent pathways enriched in quantiles with differential exosomal miRNA patterns in PD (Table S1), suggesting that these biologic pathways were involved in development of PD. The KEGG pathway “Neurotrophin signaling pathway” was significantly altered in PD patients with 13 miRNAs (miR-1, miR-331-5p, miR-153, miR-132-5p, miR-485-5p, miR-409-3p, miR-433, miR-370, let-7g-3p, miR-873-3p, miR-136-3p, miR-19b-3p, and miR-10a-5p) targeting 42 genes in the pathway map (Figure S3). The KEGG pathway “Dopaminergic synapse” was also significantly altered in PD patients with 9 miRNAs (miR-1, miR-153, miR-485-5p, miR-409-3p, miR-433, let-7g-3p, miR-136-3p, miR-19b-3p, and miR-10a-5p) targeting 41 genes in the pathway map of Dopaminergic synapse (Figure S4). The KEGG pathway “Cholinergic synapse” was significantly enriched in PD patients with 11 miRNAs (miR-1, miR-153, miR-132-5p, miR-485-5p, miR-409-3p, miR-433, miR-370, let-7g-3p, miR-873-3p, miR-19b-3p, and miR-10a-5p) targeting 40 genes in the Cholinergic synapse pathway (Figure S5).

In addition, there were six KEGG biological processes significantly enriched (*p* < 0.05, FDR corrected) among differentially expressed exosomal miRNAs in AD CSF when compared with those of healthy controls. Among them, Taurine and hypotaurine metabolism (*p* = 0.0076), B cell receptor signaling pathway (*p* = 0.0076), Neurotrophin signaling pathway (*p* = 0.0172), VEGF signaling pathway (*p* = 0.0182), p53 signaling pathway (*p* = 0.0351), Adipocytokine signaling pathway (*p* = 0.0355) were the most prominent pathways enriched in quantiles with differential exosomal miRNA patterns in AD (Table S2), suggesting that these biologic pathways were dysregulated by miRNAs and further involved in development of AD.

### Validation of miRNA array expression using independent samples

**Figure 2:**
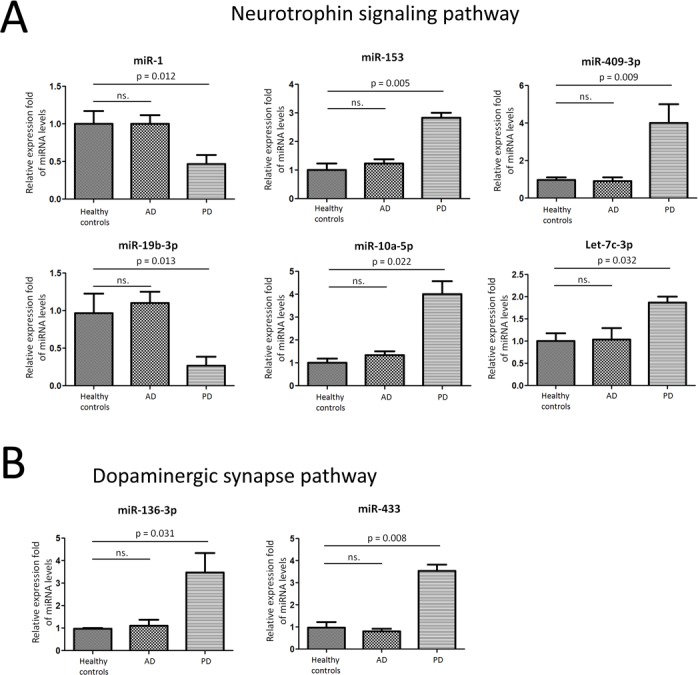
Validation of miRNA array expression using independent samples TaqMan real-time RT-PCR to validate the expression levels of **A.** Six miRNAs (miR-1, miR-153, miR-409-3p, miR-19b-3p, miR-10a-5p, and let-7g-3p) in Neurotrophin signaling pathway were selected for further validation using an independent cohort of 78 PD patients, 53AD patients, and 35 healthy controls. **B.** two miRNAs (miR-136-3p and miR-433) in Dopaminergic synapse pathway using individual CSF exosome samples. Data shown are as mean ± SEM.

We employed TaqMan Real-Time PCR to validate the expression levels of the dysregulated miRNAs from microRNA assay. The miRNAs were chosen for validation based on the significance of the difference (fold change, p-value), previous observations and biological plausibility (according to putative miRNA targets and/or Pubmed hits when particular miRNA is combined with keywords as neurodegeneration). Six miRNAs (miR-1, miR-153, miR-409-3p, miR-19b-3p, miR-10a-5p, and let-7g-3p) in Neurotrophin signaling pathway were selected for further validation using an independent cohort of 78 PD patients, 53 AD patients, and 35 healthy controls. In agreement with the preliminary data from microRNA assay, miR-1 and miR-19b-3p were significantly reduced in CSF exosome samples in PD when compared with healthy controls. While miR-153, miR-409-3p, miR-10a-5p, and let-7g-3p were significantly over expressed in CSF exosome samples (Figure 2A). In addition, we investigated another two miRNAs (miR-136-3p and miR-433) in Dopaminergic synapse pathway using individual CSF exosome samples. MiR-136-3p and miR-433 was significantly increased in PD patients (Figure 2B). Taken together, these data confirmed the validity of differentially expressed exosomal miRNAs in CSF and our results defined a profile of dysregulated CSF exosomal miRNA signature related to PD, which revealed that these miRNAs may have a functional role in the pathogenesis of PD.

### Messenger RNA transcripts and long non-coding RNAs were differentially expressed in CSF exosomes in PD and AD patients

**Figure 3:**
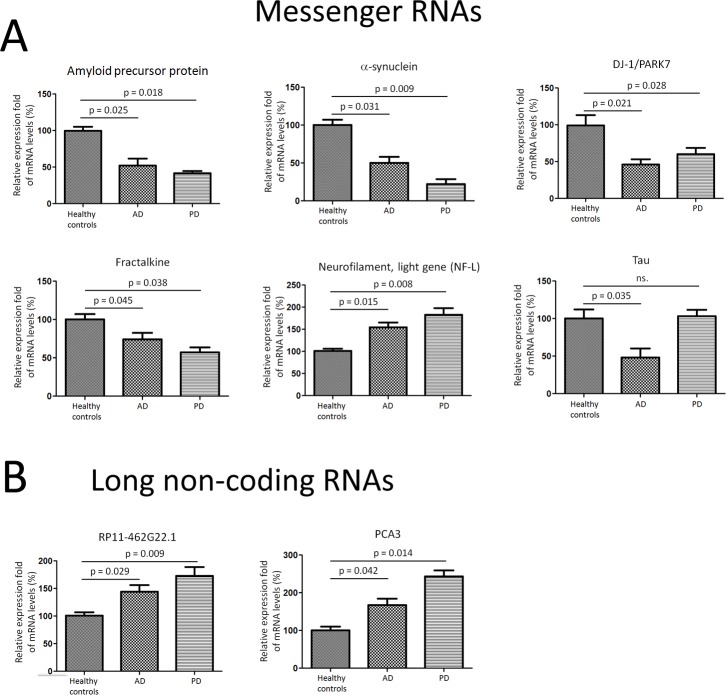
Messenger RNA transcripts and long non-coding RNAs were differentially expressed in CSF exosomes in PD and AD patients **A.** APP, α-synuclein, Tau, NF-L, DJ-1/PARK7, Fractalkine, and Neurosin, were selected for quantitative RT-PCR experiments using CSF exosomal RNA from 28 AD, 47 PD, and 27 healthy controls. **B.** Two long non-coding RNAs, RP11-462G22.1 and PCA3, were tested in CSF exosomes using quantitative RT-PCR assay. Data shown are as mean ± SEM.

In order to analyze the other components in the exosomes isolated from CSF, we tried to examine the presence of messenger RNAs (mRNAs) in exosome and elucidate their roles during development of PD and AD. The CSF exosomal RNA was heterogeneous in size. We then employed quantitative RT-PCR to detect the expression of mRNA transcripts isolated from exosome. Seven potential CSF biomarkers in PD, including amyloid precursor protein (APP), α-synuclein (α-syn), Tau, neurofilament, light gene (NF-L), DJ-1/PARK7, Fractalkine and Neurosin, were selected for quantitative RT-PCR experiments using CSF exosomal RNA from 28 AD, 47 PD, and 27 healthy controls. APP, α-syn, DJ-1, and Fractalkine were found to be present and significantly under expressed in PD and AD CSF exosome compared to healthy controls (Figure 3A). However, NF-L mRNA levels were over expressed in AD and PD CSF exosome compared to healthy controls. In addition, there were no significant changes in tau mRNA expression in PD CSF exosome, while Tau expression was significantly down regulated in AD CSF exosomes compared to healthy controls (Figure 3A).

In addition, we also observed that two PD associated long non-coding RNAs [16], RP11-462G22.1 and PCA3, were present in CSF exosome using quantitative RT-PCR assay. Both RP11-462G22.1 and PCA3 were up regulated in CSF exosome extracted from PD and AD compared to those in healthy controls (Figure 3B).

### Evaluation of CSF exosomal miRNAs as PD diagnostic biomarkers

**Figure 4:**
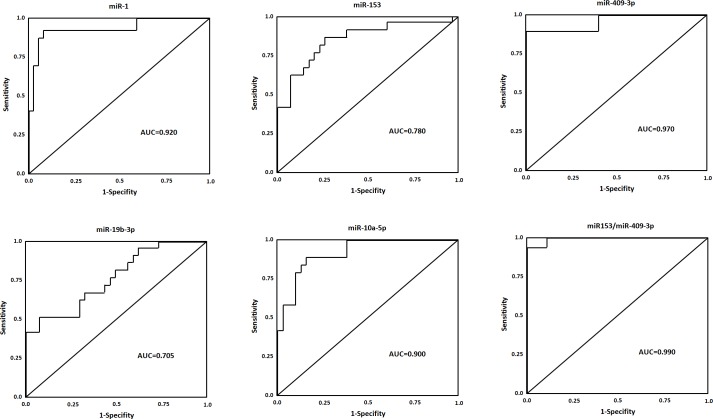
ROC curves for miRNAs that are significantly different in PD patients as compared to healthy controls ROC curve with AUC for miR-1, miR-153, miR-409-3p, miR-19b-3p, and miR-10a-5p was performed using SPSS.

To evaluate the utility of CSF miRNAs levels in discriminating cases of PD from healthy controls, ROC curve analysis was performed. We confirmed that the six miRNAs found in the primary analyses highly discriminated PD patients with healthy control (Figure 4): miR-1, AUC = 0.920, CI_95%_ = (0.845-1.014), miR-153, AUC = 0.780, CI_95%_ = (0.673-0.967), miR-409-3p, AUC = 0.970, CI_95%_ = (0.905-1.002), miR-19b-3p (AUC = 0.705, CI_95%_ = (0.643-0.917), and miR-10a-5p, AUC = 0.900, CI_95%_ = (0.772-1.063) (*p* < 0.0001 for all comparisons). The sensitivity and specificity for distinguishing Parkinson's disease from control were 94% for miR-1, 93% for miR-153, 90% for miR-409-3p, 94% for miR-19b-3p, 95% for miR-10a-5p, and 95% for let-7g-3p. Highest area under the curve (AUC) for a single miRNA could be achieved with miR-409-3p. Further the combination of miR-153 and miR-409-3p in CSF could enhance the performance of discrimination significantly (AUC = 0.990, CI_95%_ = (0.934-1.090)). These data demonstrated that exosomal miRNAs in CSF are reliable diagnostic markers for PD.

## DISCUSSION

Among several subtypes of PD defined on the basis of clinical characteristics as well as underlying neuropathology and cognitive impairment with wide variations in duration from onset of PD to the emergence of dementia onset [17, 18]. Biomarkers in CSF have also been actively investigated [8, 9, 19-22]. The most extensively studied candidate in CSF is probably α-syn, the major protein component of Lewy bodies [23]. The current consensus is that CSF α-synuclein concentrations are generally lower in PD; the sensitivity and specificity, however, appear to be only moderate, and no correlation with PD severity or progression has been observed [8, 9]. Burgos et al. provided a comprehensive examination of miRNAs detected in CSF and serum in the same patients and a comparison to reported miRNAs deregulated in AD and PD [14]. In this study we have, for the first time, verified the presence of miRNAs in CSF exosomes of PD and AD patients. Further, a substantial profile of miRNAs was differentially expressed in PD CSF exosomes. In addition, we found that aberrantly expressed messenger RNAs and long non-coding RNAs discovered in CSF exosomes may participate in the pathogenesis of PD. These results demonstrated that exosomal RNAs could discriminate PD and AD as novel cerebrospinal fluid biomarkers. Although the identified panels of CSF miRNAs could differentiate PD and AD, to fully confirm disease specificity of these potential markers, a larger cohort including related disease controls (e.g., those with multiple system atrophy or progressive supranuclear palsy) will be needed to further test their usage in PD differential diagnosis. These results, if validated independent studies, particularly those with samples collected prospectively, could be used to assist in clinical diagnosis of PD and have the potential to help monitoring or predicting disease progression.

A link between miRNAs, PD and AD is becoming increasingly evident. Dysregulated miRNAs in AD patients have been revealed using post-mortem brain samples. Profiling miRNAs from CSF in AD could lead to the identification of miRNA markers [24]. However, it should be noticed that it is far from being evident how cytoplasmic changes of miRNAs in neurons correlate with changes in the extracellular CSF. Similarly, miRNAs have been demonstrated to be associated with the dopaminergic phenotype in PD. The dysfunctional α-Synuclein has been implicated in a major mechanism in PD pathogenesis, oxidative stress might influence α-Synuclein levels via miR-7 or miR-153 inhibition [25]. Cho HJ et al., had showed that introduction of miR-205 in the neurons expressing a PD-related LRKK2 R1441G mutant prevented the neural outgrowth defects [26]. Bioinformatic methods based on sequence similarities between targets and miRNAs were used to predict the potential target genes. In the present study, we utilized DIANA-mirPath to demonstrate that Neurotrophin signaling pathway, mTOR signaling pathway, Ubiquitin mediated proteolysis, Long-term potentiation, Axon guidance, Cholinergic synapse, Gap junction, Dopaminergic synapse, Glutamatergic synapse were the most prominent pathways enriched in quantiles with differential exosomal miRNA patterns related to development of PD and AD.

Exosomes are released from the cell when multivesicular bodies fuse with the plasma membrane [27]. The origin of the exosome is the way to evaluate the importance and function of the altered miRNAs in pathogenesis of PD and AD. However, specific markers of cellular origin are not yet available. Known exosomal surface markers, such as CD63, CD81, and CD9, are primarily used for evaluating the exosomal content of the preparation. The presence of CD63 was detected in all samples in the current study, and previous investigations demonstrated the purity of exosomes isolated by using the presence of CD63 by means of FACS. It would be important to study the origin of exosomes in CSF of PD and AD patients. Numerous investigations on the role that exosome may play in cell-to-cell signaling, hypothesizing that because exosomes can merge with and release their contents into cells that are distant from their cell of origin, they may influence processes in the recipient cell [11, 28]. Conversely, exosome production and content may be influenced by molecular signals received by the cell of origin. Currently, there are no proven mechanisms by which exosomes trigger intercellular communication in the process of neurodegeneration.

There are some limitations in our study. First, a major limitation of this study is the normalization. It is not clear for reference control small RNAs in exosome study, and RNU6B or RNU44 should be used with caution, because in some circumstances normalizing by RNU6B or RNU44 in miRNA expression studies may produce misleading results. Secondly, microRNAs abundance and stability in human brain have short half-lives - many of them have half-lives of 2-3 hours or less [29]. Thus questions on the ‘down-regulated’ microRNAs we demonstrated correlated with a strong ‘degenerative’ pathology as well as drug medications effect on the exosomic microRNA profiles should be further studied.

To conclude, our study revealed a substantial abundance of miRNAs, mRNA transcripts and long non-coding RNAs present in CSF exosome of PD and AD patients. These RNA molecules were differentially expressed in CSF exosome in PD patients, and exosomal miRNAs in CSF could be evaluated as PD reliable diagnostic biomarkers. These results provide evidence for the potential value of these CSF biomarkers for the diagnosis and assessment of heterogeneous disease progression in PD and suggest dysregulation of exosome RNAs abundance for the possible recognition of prodromal PD.

## MATERIALS AND METHODS

### Participants and CSF sample collection

Written informed consent for participation in the study was obtained from either directly or from his or her guardian in all subjects and the work received approval from the institution ethics committee of Sir Run Run Shaw Hospital Affiliated with School of Medicine, Zhejiang University and in accordance with the tenets of the Declaration of Helsinki. All individuals provided informed consent, and underwent an evaluation that consisted of medical history, physical and neurological examinations, laboratory tests, and neuropsychological assessments. For discovery, 27 healthy controls, 28 AD, and 47 PD patients (a total of 102 subjects) were included in this investigation. Healthy control subjects were community volunteers in good health. They had no signs or symptoms suggesting cognitive impairment or neurological disease. All AD and PD subjects received a standard neurological examination as well as a psychiatric interview. AD patients were diagnosed with probable AD according to NINDS-ADRDA criteria [30]. The clinical diagnosis of PD was confirmed by the senior neurologist specializing in movement disorders according to UK Parkinson's disease society brain bank clinical diagnostic criteria [31]. Demographic information is listed in Table 1 for all subjects.

Another validation group of 78 patients with sporadic PD was collected from Henan province, Central China. There were 41 male and 37 female sporadic patients with mean age of 64±12 years. All PD patients underwent a standardized neurological examination by two movement disorder specialists. Fifty-three AD patients were recruited and diagnosed with probable AD according to NINDS-ADRDA criteria. A control group of 35 healthy controls from the same geographic areas was obtained. All healthy controls were free of symptoms suggestive of AD and PD, and with a negative family history of movement disorders. Informed consent was obtained before participation into our study and the work received approval from the institution ethics committee from Zhengzhou University School of Medicine in Henan province and conformed to the tenets of the Declaration of Helsinki.

### CSF samples collection

All CSF samples were obtained by lumbar puncture in the morning as described [32, 33]. Similar CSF collection protocols and quality control procedures were followed at two participating centers, in particular, use of polypropylene collection and storage tubes, rapid separation into single use aliquots, and freezing of CSF samples, to minimize potential site variations. Alpha-synuclein protein, tau protein, DJ-1 protein levels in CSF of all subjects were examined using Luminex assay according to previous studies [32, 33].

### Isolation of exosomes

Exosomes were isolated as described before [34]. In brief, CSF was subjected to successive centrifugations of 3,000 × g (15 min) and 10,000 × g (30 min). Exosomes were then pelleted at 50,000 × g for 1 hour, using an SW28 rotor (Beckman, Brea, CA). Exosomes pellets were resuspended in 0.32 M sucrose and centrifuged at 100,000 × g for 1 h (SW60Ti rotor; Beckman Coulter Instruments), and then resuspended in PBS for −80°C storage.

### Exosome characterization by FACS analyses

CSF was added directly to Dynabeads (2 mL/mL beads) coated with anti-CD63 antibody as previously described [35]. Beads were labeled with fluorescein isothiocyanate-labeled anti- CD63, MHC class I, anti-MHC class II, CD86 and phycoerythrin-labeled anti-CD54 or isotype-matched controls (BioLegend, San Diego, CA). Samples were analyzed in a FACS Calibur (BD Biosciences, San Jose, CA) by using forward scatter/side scatter bead gating, and mean fluorescence intensity (MFI) ratios were calculated as the geometric mean of the marker divided by the geometric mean of the isotype control.

### Electron microscopy

Exosomes from CSF were loaded onto formwar carbon-coated grids, fixed in 2% paraformaldehyde, washed and immunolabelled with anti-CD63 antibody followed by 10 nm gold-labelled secondary antibody (Sigma Aldrich). The exosomes were post-fixed in 2.5% glutaraldehyde, washed, contrasted in 2% uranyl acetate, embedded in a mixture of uranyl acetate (0.4%) and methyl cellulose (0.13%), and examined by electron microscope (Carl Zeiss NTS).

### RNA processing and miRNA profiling

Exosomal RNA was extracted using the Qiagen miRNeasy Serum/Plasma Kit (Qiagen, Valencia, CA) according to the manufacturer's instructions. RNA quality was assessed by using UV 260/280 and 230/260 absorbance ratios. RNA size distribution was examined on RNA Pico LabChips (Agilent Technologies, Palo Alto, CA) processed on the Bioanalyzer (Agilent). TaqMan Low-Density Array Human miRNA Panels (Applied Biosystems, Foster City, CA) were utilized for global miRNA profiling. The panel includes two 384-well microfluidic cards (human miRNA pool A and pool B) that contain primers and probes for 746 different human miRNAs in addition to six small nucleolar RNAs that function as endogenous controls for data normalization. 25 ng RNA was reverse transcribed for cDNA synthesis using the TaqMan Multiplex RT set (Applied Biosystems) for TaqMan Array Human MicroRNA Panels. Quantitative PCR was performed on an Applied BioSystems 7900HT thermocycler (Applied Biosystems) using the manufacturer's recommended cycling conditions. The relative expression levels between samples were calculated using the comparative delta Ct (threshold cycle number) method [36].

### MiRNA target prediction and pathway analysis

DIANA-mirPath [37] was used to perform target prediction and pathway analysis based on two algorithms, microT-CDS [38, 39] and miRTarBase [40]. The software performs an enrichment analysis of multiple miRNA target genes to Kyoto Encyclopedia of Genes and Genomes (KEGG) pathways. The graphical output of the program provides an overview of the parts of the pathway modulated by selected miRNAs. The statistical significance value associated with the identified biological pathways was calculated by mirPath.

### TaqMan miRNA Assay for Individual miRNAs

Independent sets of exosome samples from CSF were used for qPCR confirmation. The selected differentially expressed miRNAs from miRNA array discovery panels were further quantitated by TaqMan miRNA assays (Applied Biosystems).

### Quantitative real-time PCR (QPCR)

Candidate mRNA transcripts levels were measured by real-time PCR (SYBR Green Supermix; Applied Biosystem) using the thermo-cycler (Bio-Rad, Hercules, CA). Melting curve analysis was done at the end of the reaction to assess the quality of the final PCR products. The threshold cycle C(t) values were calculated by fixing the basal fluorescence at 0.05 units. The N-fold increase or decrease in expression was calculated by the ΔΔCt method using the C(t) controls value as the reference point.

### Statistical analysis

Data analysis on miRNA expression levels from TaqMan Low-Density Array was performed by SDS software version 2.2.2 (Applied Biosystems). Assays that had Ct values >35 were removed from the analysis. The delta Ct values were calculated by using RNU44 and RNU6B as the endogenous controls. A univariate test to screen for differentially expressed miRNAs since we initially designed the experiments to minimize the impact of covariates by matching the samples for key confounding factors. Bonferroni procedure was used to calculate adjusted p values to control false discovery rate (FDR) among groups and different normalizers. Heatmap of differentially expressed miRNAs and between-group statistic analysis were performed by R software. A receiver operating characteristic (ROC) curve was performed to calculate the relationship between sensitivity and specificity for disease group versus healthy controls. Data analysis and ROC curve analysis was performed using SPSS version and GraphPad Prism. All data were expressed as mean ± SEM. P values less than 0.05 were considered statistically significant.

## SUPPLEMENTARY MATERIAL FIGURES AND TABLES


