# Supplementary material for: Altered microRNA profiles in cerebrospinal fluid exosome in Parkinson disease and Alzheimer disease
Source: Oncotarget. 2015 Oct 19;6(35):37043–53. doi: 10.18632/oncotarget.6158 (PMC4741914; doi:10.18632/oncotarget.6158)
Supplement: Supplementary file 1 [file oncotarget-06-37043-s001.pdf]

Altered microRNA profiles in cerebrospinal fluid exosome in Parkinson disease and Alzheimer disease

Supplementary Material

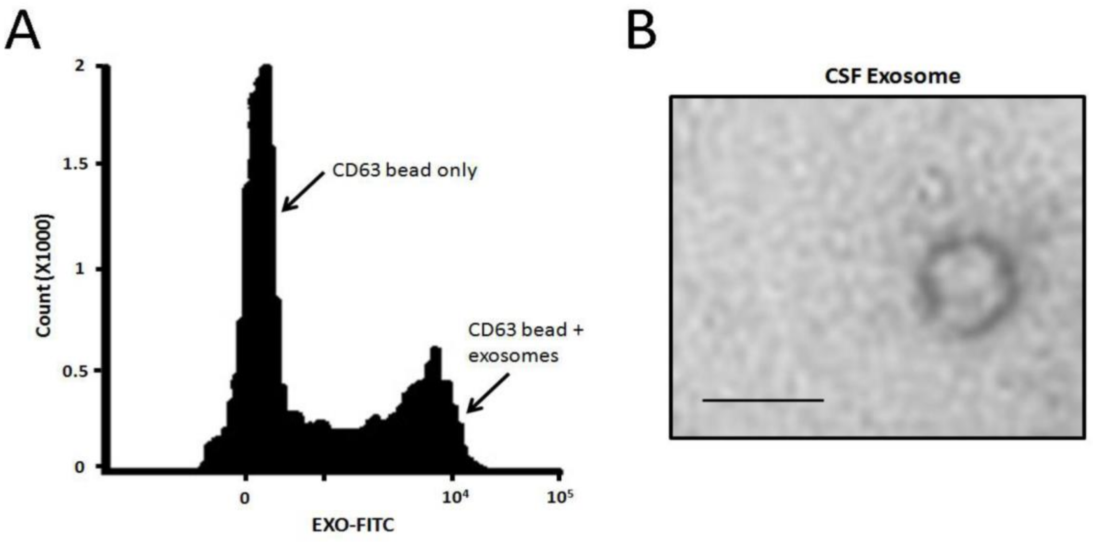

Figure S1. Identification and characterization of CSF exosomes. (A). Flow cytometric analysis of CSF exosomes displayed expression of CD63 surface markers. Results are shown as the MFI for the detected molecule divided by the MFI for the isotype control. Data shown are representative of at least three independent experiments. (B). Electron micrographs of CSF exosomes. The scale bar indicates 100 nm.

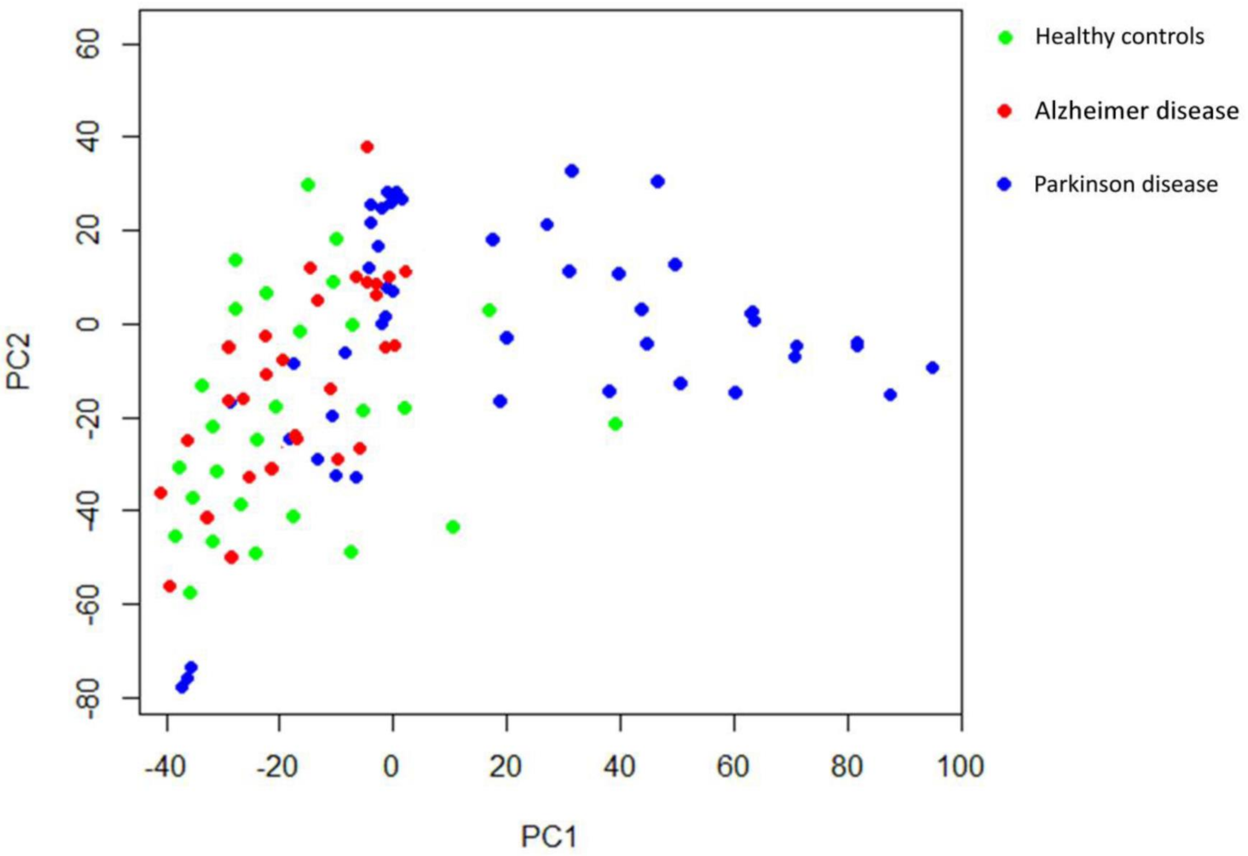

Figure S2. Principal component analysis.

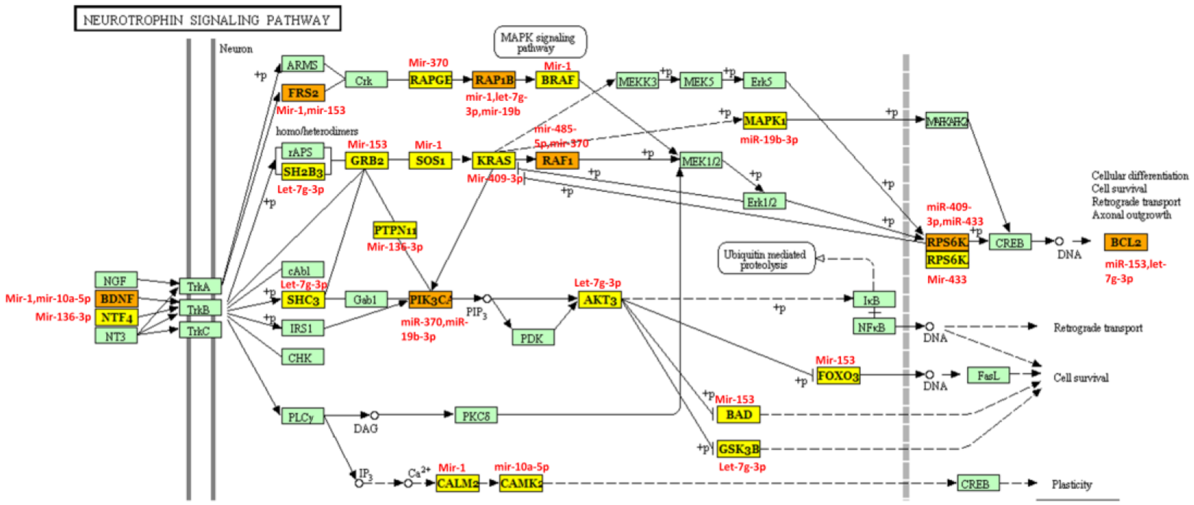

Figure S3. The KEGG pathway "Neurotrophin signaling pathway" was significantly altered between PD patients and healthy subjects at baseline, with 13 miRNAs (hsa-miR-1, hsa-miR-331-5p, hsa-miR-153, hsa-miR-132-5p, hsa-miR-485-5p, hsa-miR-409-3p, hsa-miR-433, hsa-miR-370, hsa-let-7g-3p, hsa-miR-873-3p, hsa-miR-136-3p, hsa-miR-19b-3p, hsa-miR-10a-5p) targeting 42 genes. Targeted genes are indicated by yellow (one miRNA) and brown (multiple miRNAs) colors in the pathway map.

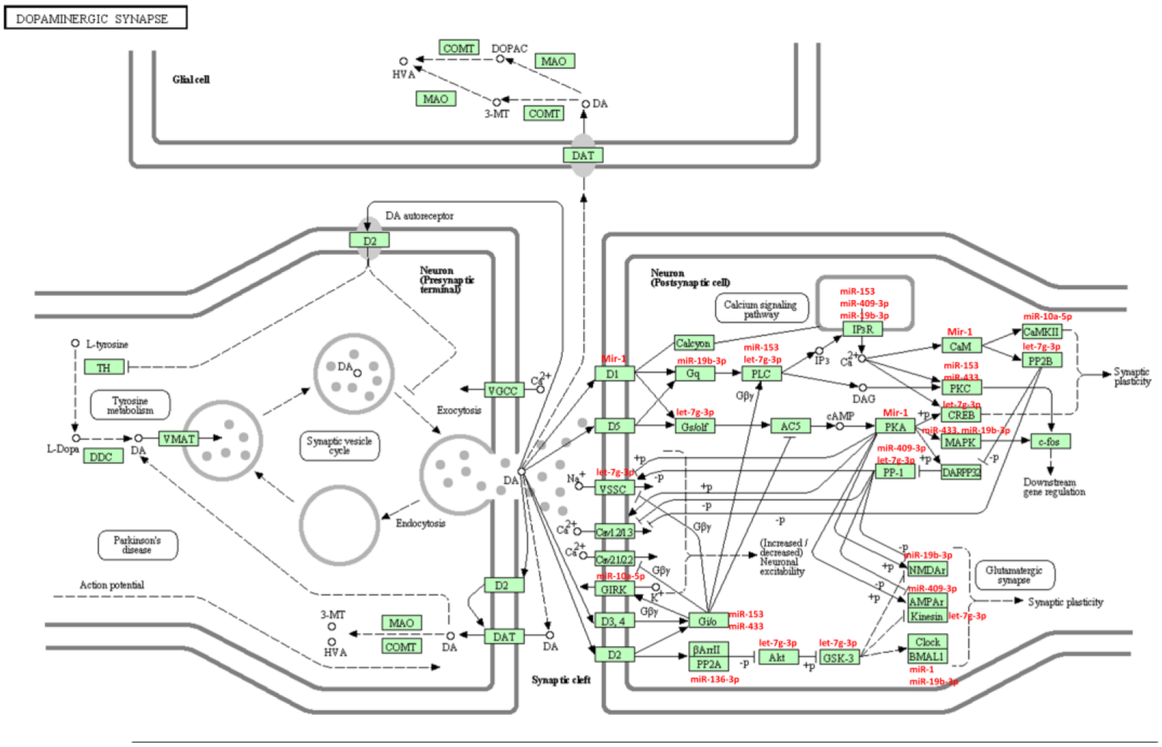

Figure S4. The KEGG pathway "Dopaminergic synapse" was significantly altered between PD patients and healthy subjects at baseline, with 9 miRNAs (hsa-miR-1, hsa-miR-153, hsa-miR-485-5p, hsa-miR-409-3p, hsa-miR-433, hsa-let-7g-3p, hsa-miR-136-3p, hsa-miR-19b-3p, hsa-miR-10a-5p) targeting 41 genes. Targeted genes are indicated by miRNAs (Red colors) in the pathway map.

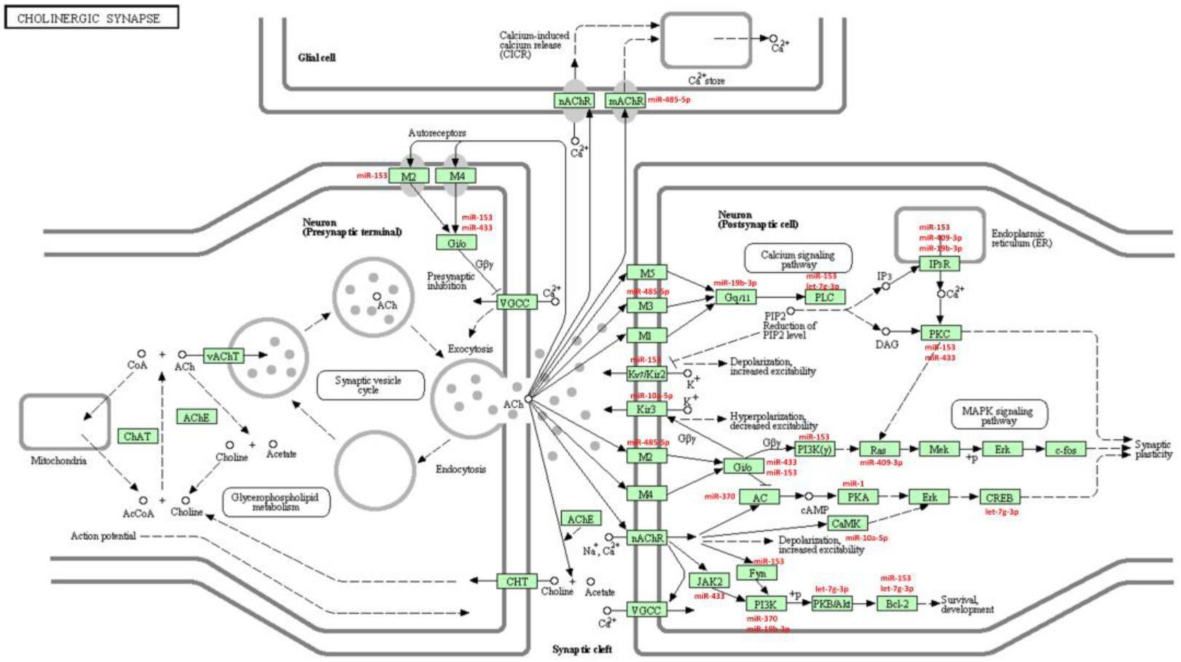

Figure S5. The KEGG pathway "Cholinergic synapse" was significantly altered between PD patients and healthy subjects at baseline, with 11 miRNAs (hsa-miR-1, hsa-miR-153, hsa-miR-132-5p, hsa-miR-485-5p, hsa-miR-409-3p, hsa-miR-433, hsa-miR-370, hsa-let-7g-3p, hsa-miR-873-3p, hsa-miR-19b-3p, hsa-miR-10a-5p) targeting 40 genes. Targeted genes are indicated by miRNAs (Red colors) in the pathway map.

Table S1. Biologic pathways enriched by differential exosomal miRNAs in CSF in PD patients.

# KEGG pathway	KEGG pathway	p-value, FDR corrected	#genes	#miRNAs	Prediction
hsa04910	Insulin signaling pathway	9.68E-16	48	12	microT-CDS
hsa04722	Neurotrophin signaling pathway	5.70E-13	42	13	microT-CDS
hsa04150	mTOR signaling pathway	2.86E-12	26	11	microT-CDS
hsa04120	Ubiquitin mediated proteolysis	2.86E-12	46	13	microT-CDS
hsa04720	Long-term potentiation	3.68E-12	27	10	microT-CDS
hsa04151	PI3K-Akt signaling pathway	5.62E-12	89	12	microT-CDS
hsa04510	Focal adhesion	6.42E-12	60	12	microT-CDS
hsa04360	Axon guidance	3.51E-11	43	11	microT-CDS
hsa04725	Cholinergic synapse	1.75E-10	40	11	microT-CDS
hsa04540	Gap junction	7.09E-10	29	9	microT-CDS
hsa04960	Aldosterone-regulated sodium reabsorption	1.03E-08	16	10	microT-CDS
hsa04728	Dopaminergic synapse	3.43E-08	41	9	microT-CDS
hsa04115	p53 signaling pathway	3.43E-08	24	10	microT-CDS
hsa04310	Wnt signaling pathway	5.15E-08	47	11	microT-CDS
hsa04012	ErbB signaling pathway	7.35E-08	29	11	microT-CDS
hsa00430	Taurine and hypotaurine metabolism	6.70E-07	6	6	microT-CDS
hsa04370	VEGF signaling pathway	9.07E-06	21	10	microT-CDS
hsa04062	Chemokine signaling pathway	1.17E-05	48	11	microT-CDS
hsa04724	Glutamatergic synapse	1.76E-05	31	10	microT-CDS
hsa03018	RNA degradation	3.17E-05	22	9	microT-CDS
hsa04520	Adherens junction	4.38E-05	23	8	microT-CDS

hsa04920	Adipocytokine signaling pathway	8.09E-05	20	10	microT-CDS
hsa04810	Regulation of actin cytoskeleton	0.0001	53	12	microT-CDS
hsa04912	GnRH signaling pathway	0.0001	25	11	microT-CDS
hsa04320	Dorso-ventral axis formation	0.0002	9	7	microT-CDS
hsa04070	Phosphatidylinositol signaling system	0.0006	23	10	microT-CDS
hsa04961	Endocrine-regulated calcium reabsorption	0.0007	17	10	microT-CDS
hsa03015	mRNA surveillance pathway	0.0014	24	11	microT-CDS
hsa00310	Lysine degradation	0.0046	13	9	microT-CDS
hsa04350	TGF-beta signaling pathway	0.0057	22	11	microT-CDS
hsa04020	Calcium signaling pathway	0.0058	41	12	microT-CDS
hsa00471	D-Glutamine and D-glutamate metabolism	0.0059	2	2	microT-CDS
hsa00780	Biotin metabolism	0.0059	1	1	microT-CDS
hsa04973	Carbohydrate digestion and absorption	0.0073	13	9	microT-CDS
hsa04010	MAPK signaling pathway	0.0073	57	13	microT-CDS
hsa04144	Endocytosis	0.0095	46	11	microT-CDS
hsa00533	Glycosaminoglycan biosynthesis	0.0099	6	5	microT-CDS
hsa04340	Hedgehog signaling pathway	0.0173	13	8	microT-CDS
hsa04962	Vasopressin-regulated	0.0267	13	6	microT-CDS

	water reabsorption				
hsa04964	Proximal tubule bicarbonate reclamation	0.0356	7	7	microT-CDS
hsa00250	Alanine, aspartate and glutamate metabolism	0.0489	9	5	microT-CDS
hsa04210	Apoptosis	0.0493	22	10	microT-CDS

Table S2. Biologic pathways enriched by differential exosomal miRNAs in CSF in AD patients.

# KEGG pathway	KEGG pathway	p-value, FDR corrected	#genes	#miRNAs	Prediction
hsa00430	Taurine and hypotaurine metabolism	0.0076	2	2	microT-CDS
hsa04662	B cell receptor signaling pathway	0.0076	6	2	microT-CDS
hsa04722	Neurotrophin signaling pathway	0.0172	7	4	microT-CDS
hsa04370	VEGF signaling pathway	0.0182	5	2	microT-CDS
hsa04115	p53 signaling pathway	0.0351	4	1	microT-CDS
hsa04920	Adipocytokine signaling pathway	0.0355	5	2	microT-CDS
